# Enhancing Gas Fermentation Efficiency via Bioaugmentation with *Megasphaera sueciensis* and *Clostridium carboxidivorans*

**DOI:** 10.3390/bioengineering12050470

**Published:** 2025-04-29

**Authors:** Clemens Hiebl, Dominik Pinner, Hannes Konegger, Franziska Steger, Dina Mohamed, Werner Fuchs

**Affiliations:** 1 Department of Agricultural Sciences, Institute of Environmental Biotechnology, University of Natural Resources and Life Sciences, Vienna, Konrad-Lorenz-Strasse 20, 3430 Tulln an der Donau, Austria; clemens.hiebl@boku.ac.at (C.H.); dominik.pinner@outlook.com (D.P.); hannes.konegger@boku.ac.at (H.K.); franziska.steger@boku.ac.at (F.S.); 2 Institute of Chemical Technologies and Analytics, TU Wien, Getreidemarkt 9, 1060 Vienna, Austria

**Keywords:** *Megasphaera sueciensis*, *Clostridium carboxidivorans*, bioaugmentation, mixed microbial culture, gas fermentation, medium-chain volatile fatty acids

## Abstract

Gas fermentation aims to fix CO_2_ into higher-value compounds, such as short or medium-chain fatty acids or alcohols. In this context, the use of mixed microbial consortia presents numerous advantages, including increased resilience and adaptability. The current study aimed to improve the performance of an enriched mixed microbial population via bioaugmentation with *Megasphaera sueciensis* and *Clostridium carboxidivorans* to improve the metabolite spectrum. The initial fermentation in trickle-bed reactors mainly yielded acetate, a low-value compound. Introducing *M. sueciensis*, which converts acetate into higher-chain fatty acids, shifted production toward butyrate (up to 3.2 g/L) and caproate (1.1 g/L). The presence of *M. sueciensis* was maintained even after several media swaps, showing its ability to establish itself as a permanent part of the microbial community. Metataxonomic analysis confirmed the successful integration of *M. sueciensis* into the mixed culture, with it becoming a dominant member of the Veillonellaceae family. In contrast, bioaugmentation with *C. carboxidivorans* was unsuccessful. Although this strain is known for producing alcohols, such as butanol and hexanol, it did not significantly enhance alcohol production, as attempts to establish it within the microbial consortium were unsuccessful. Despite these mixed results, bioaugmentation with complementary microbial capabilities remains a promising strategy to improve gas fermentation efficiency. This approach may enhance the economic feasibility of industrial-scale renewable chemical production.

## 1. Introduction

Elevated atmospheric CO_2_ levels represent a significant threat, driving global warming and resulting in rising sea levels, altered weather patterns, and profound impacts on ecosystems [1,2]. Renewable fuels are crucial for climate change mitigation by reducing greenhouse gas emissions from transportation and energy sectors, offering a sustainable alternative to fossil fuels [3]. Gas fermentation is regarded as an emerging biotechnological tool with significant potential to contribute to the production of renewable chemicals and fuels [4,5,6]. Fermentable gaseous substrates can be derived from CO_2_ captured from off-gases in combination with H_2_ produced via electrolysis of surplus electricity [7]. Alternatively, syngas, a mixture of H_2_, CO, and CO_2_, can be utilized [8]. Syngas is typically generated through the anoxic thermal decomposition of organic matter. The fermentative process serves as a biochemical alternative to the chemo–catalytic route, commonly known as Fischer–Tropsch synthesis [9,10,11]. CO_2_ fixation in the form of acetate represents only the initial step. The ultimate goal is to generate more valuable products with higher molecular weights, such as volatile fatty acids (VFAS) or alcohols [12]. Chain elongation, i.e., the elongation of the carbon backbone, is the key biochemical process for achieving this objective. It is a growth-dependent anaerobic metabolism that combines acetate and ethanol to produce butyrate, hexanoate, and octanoate [13]. While the microbial conversion of such gas mixtures has primarily been investigated using pure cultures, several other studies highlight the potential advantages of employing mixed microbial consortia. In nature, microbes rarely live in isolation; rather, they exist in diverse and complex communities. Microbes in these communities interact in various ways, often performing complex tasks that single species cannot accomplish independently. Hence, mixed microbial cultures are well suited to conduct carbon capture and chain elongation in a single bioreactor system [14]. There are rapidly growing efforts to understand natural consortia and to engineer synthetic consortia for biotechnology applications [15,16]. Well-engineered microbial consortia involving several species can take advantage of the functions of individual microbes and their interactions to realize synergistic division of labor and more efficient utilization of biochemical substrates than monocultures. In addition to the ability to perform advanced biosynthetic tasks, microbial consortia exhibit appealing properties, including enhanced resilience and adaptive capacity, as well as the ability to operate in non-sterile conditions, all of which contribute to reducing the overall financial costs compared to pure culture-based processes [17]. These benefits are particularly well suited to the requirements for successful industrial-scale syngas fermentation, which include flexible operation with various feedstocks, a robust process capable of handling disturbances arising from syngas quality variability and the presence of inhibitors, as well as improvements in productivity and selectivity, all while ensuring efficient utilization of syngas components at reduced capital and operating costs [14].

In recent years, studies focusing on chain-elongating cultures have primarily concentrated on species from the class Clostridia [18]. However, other potential chain elongators, such as *Megasphaera* spp., have been isolated and warrant further investigation [19]. Evidence suggesting that the strain *Megasphaera sueciensis* may also be effectively employed for chain elongation has been presented by Batlle-Vilanova and co-workers [19]. Additionally, batch pre-experiments conducted in our laboratory supported this hypothesis, as the formation of butyrate from acetate was observed. In a parallel experimental approach, continuous gas fermentation was established in trickle-bed reactors using mixed microbial cultures. The microbial cultures originally derived from a biogas plant and were adapted to the acetogenesis through long-term gas fermentation. The predominant product of the established mixed culture was acetate, a low-value compound, albeit produced in high concentrations of up to 700–800 mmol/L. Results from these experiments have been published previously [20]. In the current paper, we report on the attempt to enhance the formation of short- to medium-chain products through the bioaugmentation of the chain-elongating strain *M. sueciensis* and *C. carboxidivorans* to an already established mixed culture.

## 2. Materials and Methods

### 2.1. Experimental Setup

**Figure 1:**
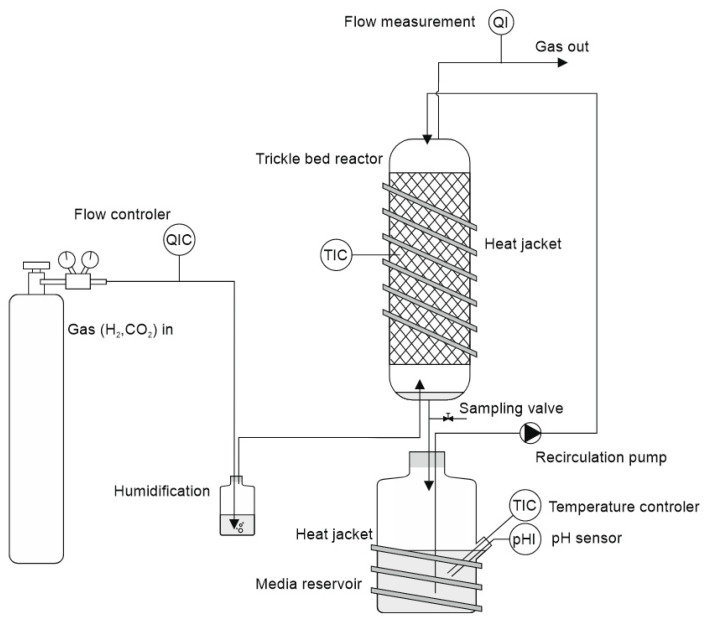
Scheme of the trickle-bed reactor setup employed in the experiments.

A scheme of the experimental set-up is provided in Figure 1. The columns were made of borosilicate glass cylinders with an inner diameter of 38.1 mm and a length of 430 mm. The columns were filled with 343 carriers (Bioflow 9’’ PE black, 800^2^/m^3^, RTV Process Equipment GmbH, Marktrodach, Germany) and the filter bed height was 33.5 mm, resulting in an empty bed volume of ca. 380 mL. The ends of the glass columns were sealed with screw caps (GL45, DURAN Group, Mainz, Germany), which contained a lid element with three quick connectors for attaching tubing (PUN-H-6x1, FESTO, Esslingen, Germany) to allow gas entry/exit and medium circulation. From the bottom of the column, the media (total volume 400 mL) drained into a one-liter Schott flask, which served as the media reservoir. From there, the media was recirculated to the top using a multichannel peristaltic pump (PD 5201, Heidolph, Schwabach, Germany) at a rate of 9.8 mL min^−1^. Gaseous substrate (H_2_:CO_2_ = 80:20, Linde, Austria) was supplied in counterflow from a pressurized gas cylinder at a flow rate of 7 mL/min employing a mass flow controller (MFC, type 8711, Bürkert, Ingelfingen, Germany). It entered the packed column from the bottom; after exiting the outlet at the top, the gas passed through a volumetric gasometer (RITTER MilliGascounter MGC-1 PMMA, Ritter, Bochum, Germany), where the residual gas flow was measured. Finally, the gas was released into the environment and removed by a fume hood. Each of the four reactors had a separate media recirculation and gas flow control. Temperature sensors in the jacket (PRO PT100 RTD Sensor, RS Components, RS Components, Corby, UK) and in the reservoir tank (combined pH and temperature sensor), along with heating jackets (RS PRO silicone heater mat, RS Components, UK), were employed to maintain a constant temperature of 30 °C throughout the system. The pH was adjusted to a fixed starting point at the beginning of each sample period (2–3 days). At each period, the pH was readjusted to the starting point using NaOH (1.0 M). Due to the high concentration of the added alkali, the delayed pH adjustment caused by its reaction with dissolved CO_2_ or HCO_3_^−^, and the slow equilibration between the medium reservoir and the trickle-bed reactor, slight deviations occurred in maintaining the setpoint. Typically, the pH dropped from 6 to 6.5 to values of 5.0 to 5.5, depending on the formation of organic acids. A very recent study also employed mixed cultures using a very similar experimental setup; however, it included a pH control system that allowed operation at a fixed set point [21]. Five pH values were tested in the range of 4.5–7.5, and pH 6 was found to be the most favorable for the simultaneous production of butyrate and caproate from syngas. At pH 5.3, the chain elongation yield in the target products dropped sharply and continued decreasing at pH 4.5.

### 2.2. Microorganisms

*Megasphaera sueciensis* (DSMZ 17042) is a gram-negative, anaerobic, non-motile, non-spore-forming diplo-cocci strain from the *Veillonellaceae* family. It was isolated from rotten Swedish beer and is able to metabolize ethanol and acetate into longer chain carboxylic acids using the reverse beta oxidation pathway. Its temperature growth lies between 15 and 37 °C with an optimum at 30 °C [22]. Its main products from PYE media are butyric acid, isovaleric acid and valeric acid.

*Clostridium carboxidivorans* (DSMZ 15243) is a gram-positive, anaerobic, motile, and spore-forming rod-shaped bacteria. It converts CO_2_, CO, and H_2_ into acetate and is able to elongate the carbon chain up to caproic acid and also to form the respective alcohols. Its temperature growth range lies between 24 °C and 42 °C [23] and has its optimum at 39 °C. Since the culture is capable of autotrophically producing hexanol, it has been the focus of interest in gas fermentation over the last years.

### 2.3. Media

The composition of the liquid media applied in the trickle-bed reactors is provided in Table 1. The composition of the referenced vitamin and mineral solutions are listed in Table 2 and Table 3. Prior to use, 40 mM 2-Bromoethanesulfonate (BES) was added as an inhibitor of methanogenic archaea.

### 2.4. Pre-Cultivation of M. sueciensis and C. carboxidivorans

Freeze-dried *M. sueciensis* strains were reactivated following the DSMZ protocol using complex peptone yeast extract (PYE) media. After 2–3 days of cultivation, cultures were transferred to 100 mL serum flasks containing 50 mL of media (Table 2), supplemented with 2.5 g/L NaAcetate as a carbon source and N_2_ in the headspace (1.5 bar).

Reactivation of *C. carboxidivorans* was conducted in a similar manner using complex peptone yeast extract plus salts media (PY + X) media (DSMZ Media 104c). After reaching a sufficiently high OD (typically OD > 1), 3 mL were used to inoculate the autotrophic media listed in Table 4. The composition of the used Ni-Se-W stock is provided in Table 5. H_2_ and CO_2_ in the headspace served as the electron donor and acceptor, respectively. The autotrophic culture was then centrifuged, resuspended in RO-water and added to the column. 

### 2.5. Analytical Methods

#### 2.5.1. HPLC

Concentrations of fatty acids and alcohols were determined by HPLC analysis (Agilent 1260 Infinity II Series HPLC System with G4212B Diode Array Detector, Agilent Technologies, Santa Clara, CA, USA) on an IC Sep ICE-Coregel ION 300 Column (Concise Separations, San Jose, CA, USA) with a mobile phase of 0.02 M H_2_SO_4_ at a flow rate of 0.325 mL/min. The column oven and detector temperatures were set to 45 °C.

#### 2.5.2. OD

Optical density (OD) was measured at 600 nm (spectrophotometer DR2800, Hach Lange, Düsseldorf, Germany).

#### 2.5.3. GCMS

Carboxylic acids and alcohols with longer chain lengths were analyzed using GC-MS after derivatization with sialylation to enhance volatility for GC separation. A 5 mL filtered sample was extracted with 3 mL CH_2_Cl_2_ (1 min hand shaking, 5 min sonication). The extract was filtered through Na_2_SO_4_ to remove water, then centrifuged (11,292× *g*, 1000 rpm, 10 min, 5920 R, Eppendorf, Hamburg, Germany). The filtered extract (240 µL) was combined with 80 µL O-Bis(trimethylsilyl)trifluoroacetamide (BSTFA), mixed, and incubated at 60 °C for 30 min. GC-MS analysis was performed using an Agilent 7890A GC with a Combi PAL Autosampler and a 5975C mass selective detector. A DB5-MS J&W column (30 m × 0.25 mm × 0.25 µm) was used, with a column flow of 1.2 mL/min, an injector temperature of 200 °C, and a 3:1 split ratio oven program from 60 °C (1 min hold) ramped to 325 °C (15 °C/min held for 6 min. The MS was set to scan from m/z 20 to m/z 450, operating in scan and selected ion monitoring (SIM) modes to detect alkanes, alcohols, and fatty acids.

#### 2.5.4. Metataxonomic Analysis

Cells were harvested from the liquid media by centrifugation (3068× *g*, 30 min) and subsequently subjected to three freeze–thaw cycles to induce cell rupture. The DNA was extracted using a FastDNA™ SPIN Kit for Soil (MP Biomedicals Germany GmbH, 37269 Eschwege, Germany) following the manufacturer’s instructions. The metagenome was characterized using amplicons of the 16Sv4 region of the 16S rRNA gene. Sequencing and data analysis were performed by Microsynth AG (Balgach, Switzerland). Additional details on the procedure can be found in Hummel et al., 2025 [24].

## 3. Results and Discussion

**Figure 2:**
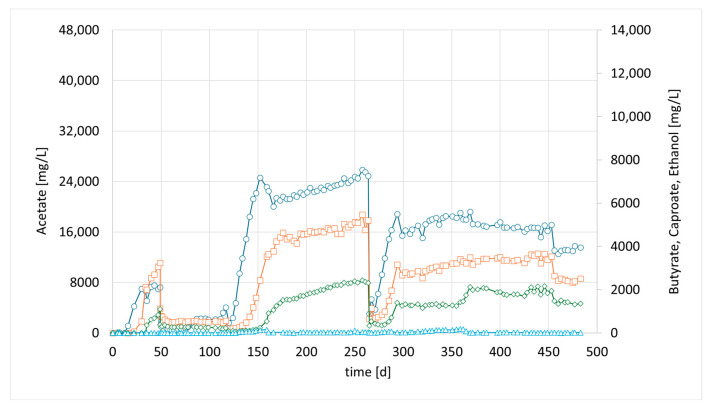
Course of metabolite concentration in CHER I; Indigo circles: acetate, orange squares: butyrate, green diamonds: caproate, blue triangles: ethanol; Key events: day 7—inoculation with *M. sueciensis*, day 49—media swap, day 119—media swap after fixing leakage; day 265—media swap. Sample for Metataxonomic analysis taken at day 315.

In the preceding syngas fermentation experiments mentioned above, a high acetate concentration of up to 40.9 g/L was achieved. However, the production of compounds with a longer chain length remained very low, with a maximum concentration of 0.93 g/L butyric acid, reaching only 2% of the main product, acetate [20]. As explained, we tried to accelerate chain elongation in the continuous culture by the addition of the strain *M. sueciensis*. For the current study, a new column was set up and inoculated with 50 mL (total media 400 mL) of a refrigerated mixed culture from the previous experiment. The intention was to revive the culture and promote attachment to the fresh carriers without allowing the development of a mature biofilm that could hinder the introduction of a new species. After an initial 7-day period, a freshly grown culture of *M. sueciensis* was added to the chain-elongating reactor I (CHER I). As illustrated in Figure 2, the formation of medium-chain acids increased quickly after adding the chain-elongating microorganism. Almost 10% of the total product was butyrate. Around 4 weeks after inoculation (day 35), even caproic acid was detected for the first time. The final levels in this first attempt were 7.2 g/L acetic acid 3.2 g/L butyric acid and 1.1 g/L caproic acid (Figure 2, at 40 days). After that, a media swap was conducted to see if the performance of the established mixed culture remains stable. To our disappointment, the initial results did not seem to be reproducible. It took a while for the reason to be discovered: a crack in a three-way sampling valve, allowing small amounts of oxygen to enter the reactor. However, after sealing the leak, high butyrate concentrations were observed once again. In this second successful phase, even higher final butyrate concentrations were achieved compared to the previous phase. After around another 150 days, another media swap was conducted. Again, high concentrations were observed, and the share of butyrate and caproate was higher, even though overall concentrations were lower than in phase 2. The typical concentration of dissolved bacteria in the liquid medium, as determined by OD, ranged from approximately 0.8 to 1.4. Lower values were observed only at the initiation of each run; however, within a few days, the concentration stabilized within this range and exhibited slight fluctuations with a gradual increase over time. Notably, the OD did not significantly affect the product spectrum or concentration, suggesting that the majority of the active microorganisms were present within the biofilm. Biofilm formation was clearly visible; unfortunately, no methods were available to quantify the total bacterial biomass within the biofilm.

**Figure 3:**
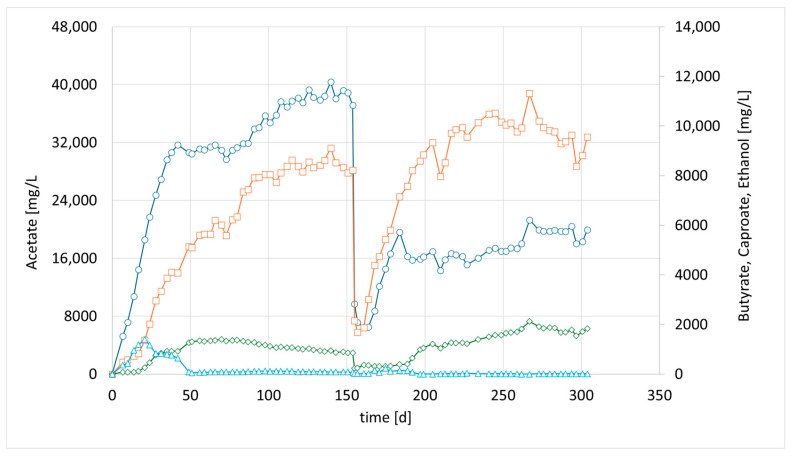
Course of metabolite concentration in CHER II; Indigo circles: acetate, orange squares: butyrate, green diamonds: caproate, blue triangles: ethanol; Key events: day 0—inoculation with mixed culture (CHER I), day 150—media swap. Sample for metataxonomic analysis taken on day 205.

Meanwhile, more bioreactors became available, which have been used for bio methanation experiments before. CHER II was established with the aim of checking whether the modified mixed culture can be transferred to a new bioreactor and to confirm the observations made in the first setup. To inoculate this second column, 50 mL of the media of the first column (CHER I) was used. The results are illustrated in Figure 3. The data showed good reproducibility of the earlier findings. After the first media swap, the ratio of butyrate to acetate shifted from 1:5 [1:4] to 1:2, reaching acetate values of 21 g/L and butyrate levels of up to 11 g/L, while caproate reached a concentration of 2 g/L. An overview of the best results achieved in the individual reactors is provided in Table 6. To contextualize the concentrations achieved in the experiments, values of up to 43 g/L of acetate have been reported in pure cultures utilizing syngas [25]. For mixed consortia, only lower concentrations have been observed, ranging from 2 to 15 g/L of acetate [26], with up to 2.3 g/L of butyrate [27].

While the observation of medium-chain metabolites was encouraging, the next goal was the formation of the carboxylic acids’ respective alcohols. Carboxylates are one of the major platform chemicals for the generation of bioalcohols, the main drop-in fuels to substitute petroleum gasoline [28]. Typically, the added value of the alcohols increases with the number of carbon atoms. For example, hexanoate/hexanol has twice the market value of ethanol [29]. Based on current scientific understanding, alcohol fermentation is facilitated at lower pH levels [21,30,31]. According to a recent review on syngas fermentation, studies on various *Clostridium* species revealed that a low pH (4.5–5.0) favors solventogenesis, whereas a higher pH range (5.0–6.0) promotes acetogenesis [32].

**Figure 4:**
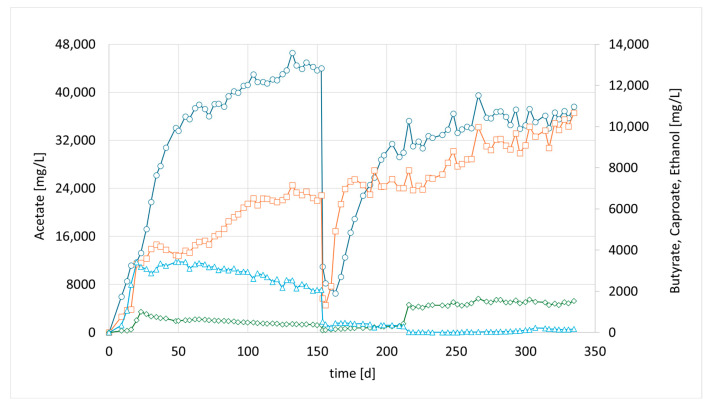
Course of metabolite concentration in CHER III; Indigo circles: acetate, orange squares: butyrate, green diamonds: caproate, blue triangles: ethanol; Key events: day 0—inoculation with mixed culture (CHER I), day 150—media swap.

Accordingly, in a third parallel experiment (CHER III), the impact of a lower pH was investigated. The pH set point of this unit was 0.5 units lower than in CHER II. An attempt was made to avoid a too low pH range as the optimum of *M. sueciensis* lies at 7.0. Typical endpoints before readjustment of pH were 5.0–5.2. As shown in Figure 4, the lowering of the pH led to elevated EtOH values that were five times higher than those observed in CHER II. However, no butanol formation was observed during these trials. CHER III best product ratios were 37.4 g/L acetate, 10 g/L butyrate and 1.6 g/L caproate at around day 255 (Figure 4). The highest measured ethanol content was 3.8 g/L. Ethanol production decreased after the first media swap, leading to an increase in butyrate concentration and an improved acetate-to-butyrate ratio.

**Figure 5:**
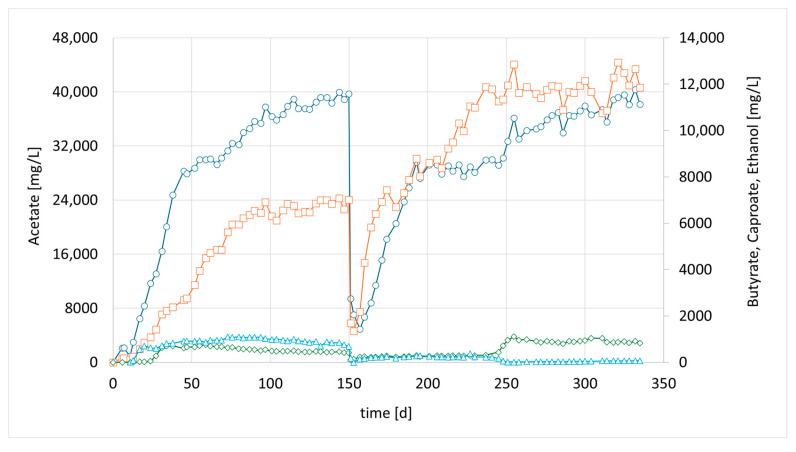
Course of metabolite concentration in CHER IV; Indigo circles: acetate, orange squares: butyrate, green diamonds: caproate, blue triangles: ethanol; Key events: day 0—inoculation with mixed culture (CHER I) + pure culture of *C. carboxidivorans*, day 150—media swap.

The final attempt, CHER IV; examined the augmentation of a second strain, the well-studied autotrophic species *C. carboxidivorans*. Similar to CHER II & III, this reactor was inoculated with a mixed culture from CHER I. After 7 days, 50 mL of a separately cultivated *C. carboxidivorans* pre-culture was added. The goal was to determine whether alcohol formation, particularly butanol production, could be enhanced. Several investigations have reported that the augmented strain is capable of producing carboxylic acids up to C6 and reducing them to their corresponding alcohols, including n-hexanol [23]. The formed products resulted in the concentrations of 36.1 g/L acetate, 12.1 g/L butyrate, and 1.0 g/L caproate (Figure 5, day 255). Alcoholic compounds were below 100 mg/L.

During this period, it was necessary to develop an analytical method for the detection and quantification of longer chain carboxylic acids/alcohols, as HPLC analysis revealed the presence of additional peaks. Due to the long retention time at the given analytical conditions, it was not feasible to retain this analysis for such products. Alternatively, the GC-MS method provided in Materials and Methods was developed. In fact, the formation of n-hexanol in CHER IV was discovered during a later stage of this fermentation (10 mg/L, around day 270). However, the concentrations of bio-alcohols in the media remained very low. As discussed further down, this low formation of hexanol cannot be clearly attributed to the augmentation of *C. carboxidivorans,* since the metataxonomic analysis showed no significant increase of the clostridia family. Overall, the metabolite profile closely resembled the performance of CHER II or CHER III, and bioaugmentation had no significant impact on the product spectrum. The new analytical method was also used to reanalyze the caprylic acid concentration in individual frozen samples taken from the reactors. The caprylic acid concentration reached 0.5 g/L in CHER I and 0.1 g/L in CHER II and CHER III, while no caprylic acid was detected in CHER IV.

### Metataxonomic Data

**Figure 6:**
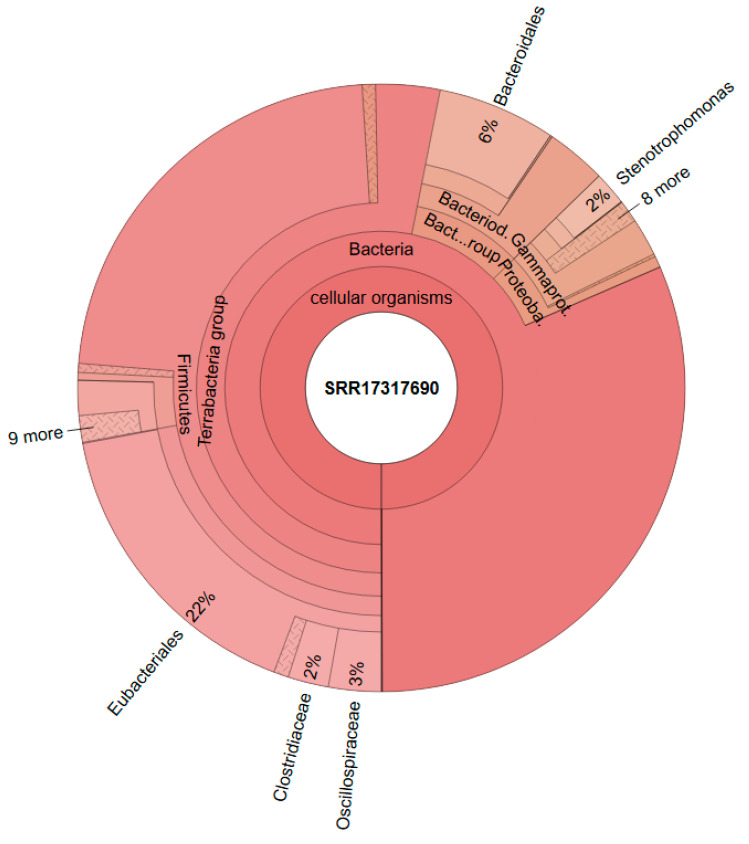
Metataxonomic analysis of the culture used as inoculum; Data from Steger et al., 2022 [20].

Figure 6 shows the composition of the consortium that was used as a starter culture for the reactors (data from Steger et al. [20]). The genus *Megasphaera* could not be detected in the inoculum, as demonstrated in Figure 6 and Supplementary Figure S1.

**Figure 7:**
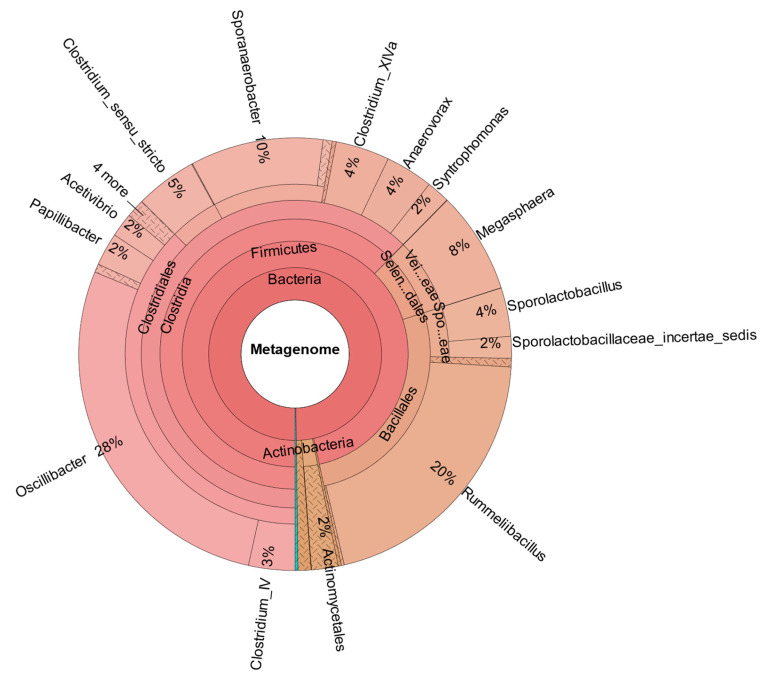
Metataxonomic analysis of CHER I at the family tree level (sample collected at day 315).

In comparison, Figure 7 shows the metataxonomic results from CHER I. The Supplementary Information provide insight in the generation of metataxonomic data for CHER I-IV, including used primer sequence and quality score. The sample was taken at day 315. As shown, *M. sueciensis* is present in a much higher share (7%) and is also the dominant member of *Veillonellales,* even though there was a time span of 144 days between inoculation with this *M. sueciensis* and sampling. This result proves that it was possible to establish the introduced species as a permanent microbial member of the mixed population. It should be underlined that all reactors were operated under non-sterile auto-selective conditions with one notable exception: the addition of the inhibitor BES in order to suppress the growth of methanogenic archaea.

**Figure 8:**
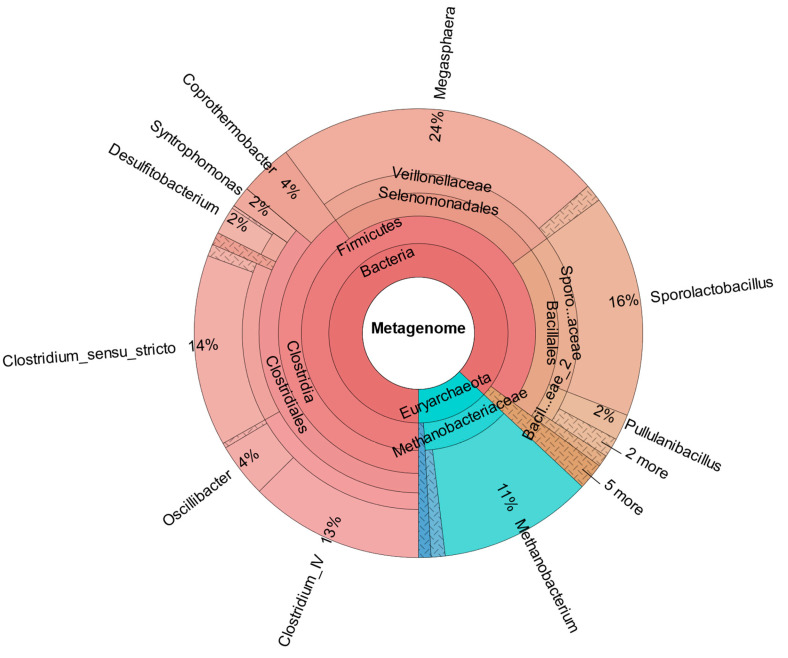
Metataxonomic analysis of CHER II at the family tree level (sample collected at day 204 of fermentation).

**Figure 9:**
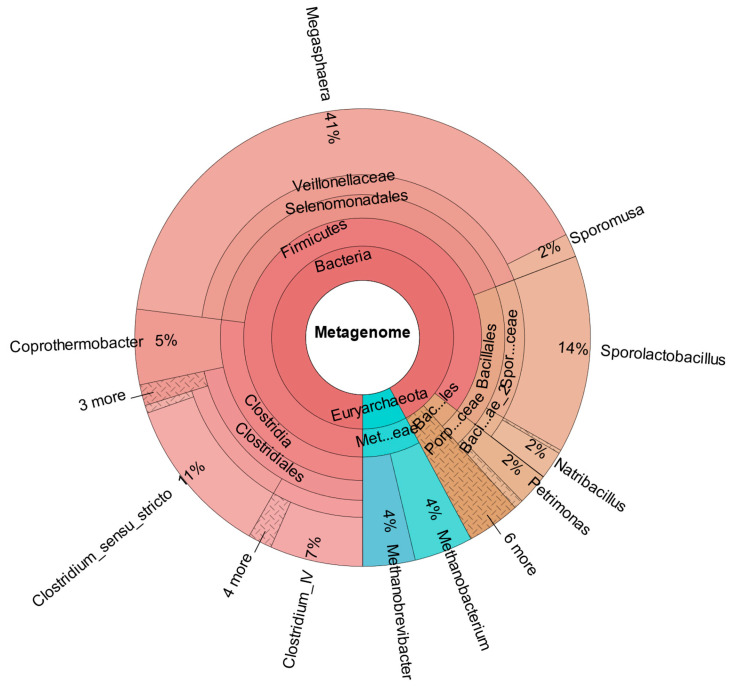
Metataxonomic analysis of CHER III at the family tree level (sample collected at day 204).

As confirmed by Figure 8, which shows the metataxonomic data of CHER II, the establishment of *M. sueciensis* is not only long lasting but it even increased in percentage proportion (24%). Unexpectedly, in this reactor, a certain share of archaeal species was also found. This is likely a remnant of the previous experiments on biomethanation of CO_2_ and H_2_. The bioreactors were flushed, and the medium was exchanged; however, sterilization was not possible. We expected that the exposure to O_2_ during reactor manipulation to prepare for the new experiment in combination with the presence of BES was sufficient to eradicate methanogenic species. Also, the permanent operation at pH levels below 6.0 must be considered. It is assumed that methanogenic species survived in niches and deeper layers of the biofilm that were not removed. Later, metataxonomic analysis confirmed that archaeal organisms declined over time. In CHER III (Figure 9), the reactor with the slightly lower pH, the proportion of *M. sueciensis* was actually the highest, despite its reported growth optimum being at neutral pH. It constitutes 96% of the identified members of the *Veillonellaceae* family.

**Figure 10:**
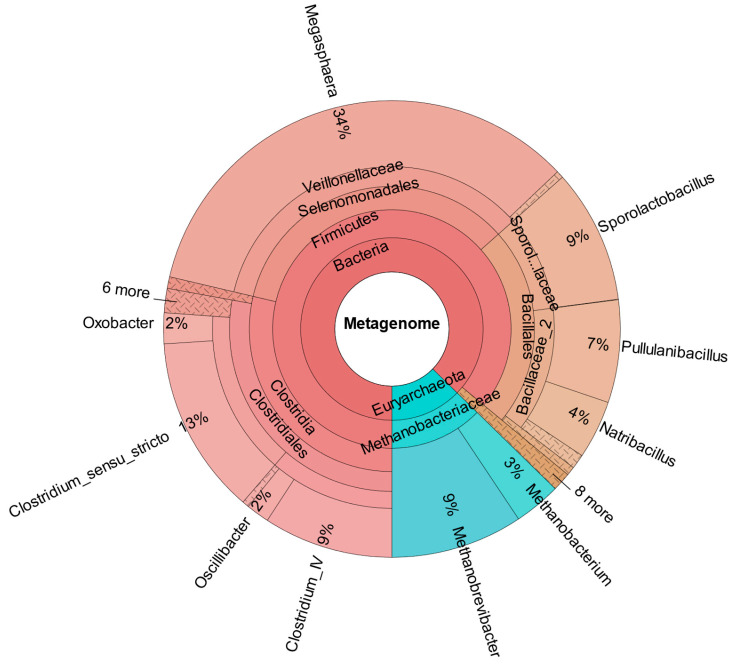
Metataxonomic analysis of CHER IV at the family tree level (sample collected at day 200).

In contrast to the results obtained, CHER IV’s metataxonomic data (Figure 10) did not indicate the successful introduction of *C. carboxidivorans*. There is a certain percentage of the parent genus *Clostridium senso stricto* (13%), although it is not significantly different from the other reactors (CHER I: 5%; CHER II: 14%, CHER III: 11%). This aligns with the low impact on product formation of this second bioaugmentation attempt.

Generally, the concept of co-cultivation of two or more defined species already has some tradition in biotechnology, e.g., co-cultivation is an established strategy for the industrial-scale fermentation of Vitamin B12 [33]. Co-cultivation has also been discussed for the enhancement of tertiary metabolite production [34]. In addition, mixed-culture fermentations utilizing enriched natural communities are a well-known strategy. In addition to its traditional application in the food industry (e.g., sourdough, yoghurt) as well as in wastewater treatment or biogas production, mixed microbial cultures have been proposed for a variety of applications in industrial biotechnology. This includes bioethanol, biohydrogen, butanol and 1,4 propanediol [35]. Several laboratory studies confirm the value of mixed microbial consortia in syngas fermentation [26,36,37]. Moreover, the authors of the abovementioned investigations [21], which closely resemble the experiments conducted here, emphasize the robustness of the system and high reproducibility of the results obtained with trickle-bed reactors.

Bioaugmentation integrates both approaches, enhancing a natural and well-adapted consortium by introducing species that provide complementary functions. Most full-scale applications are found in agriculture (particularly the introduction of nitrogen-fixing microorganisms) and soil remediation (amplifying the degradation of specific pollutants) [38]. However, there are also several examples where bioaugmentation has been used to enhance product formation. For example, Pozytek et al. list several different examples where strain addition has been applied to improve anaerobic digestion reactor stability [39] and/or biogas formation rate [40]. The generated data support the validity of this approach. The addition of *M. sueciensis* to an existing acetogenic mixed culture shifted the product spectrum from almost exclusively acetate into higher value compounds, such as butyrate, caproate and caprylate. The experiment most closely related to the current investigation, as reported in the literature, involves the bioaugmentation of a mixed bacterial culture with *Clostridium kluyveri* for the microbial production of caproate from mixed carbon sources [41]. However, in the mentioned study, the addition of *C. kluyveri*, an efficient caproic acid producer, failed to positively impact product formation. In a similar manner, our attempt to introduce a new *Clostridium* species was unsuccessful. It should be emphasized that the cultivation of the pure culture was conducted in the same growth medium and under the same gas composition as used in the trickle-bed reactor, and no indication of impaired growth was observed. One potential explanation for the failure is the presence of a relatively high number of closely related and likely well-adapted clostridial species, as revealed by the taxonomic analysis. These organisms presumably occupy the same ecological niche, which may hinder the establishment of a bacterium with a similar genetic makeup.

## 4. Conclusions

The successful utilization of enrichment cultures and their scalability has already been demonstrated in several environmental applications. A more challenging aspect of scaling up to a larger production setting is bioaugmentation, which involves the introduction of a novel species into an established microbial enrichment culture to improve product yields or specifications. This approach has been employed to modify the product spectrum in syngas fermentation, and it was immediately successful, as demonstrated by the high production of butyric acid and the sustained enhancement of chain elongation. Metabolic data confirmed the continued presence of *M. sueciensis* in high quantities within the bioreactor. However, a second, similar attempt involving *C. carboxidivorans* was unsuccessful. Together with the inconclusive findings reported in the literature, these results demonstrate that bioaugmentation to improve mixed-culture fermentations remains a largely unexplored area, with several potential pitfalls. On the other hand, the promising results obtained with *M. sueciensis* highlight that, while challenges exist, these results underscore the untapped potential of bioaugmentation in mixed-culture fermentations, warranting further research into microbial compatibility and stability. Beyond the need to identify suitable species that can integrate with the existing consortium, key research questions include the consistency of inocula when employing such manipulated mixed cultures, as well as the determination and control of population dynamics.

## Figures and Tables

**Table 1:** Media composition of continuous trials in the trickle-bed reactors.

**Species**	**Concentration [g/L]**
KH_2_PO_4_	1.000
NaCl	1.000
NH_4_Cl	0.250
MgSO_4_·7H_2_O	0.211
KCl	0.100
CaCl_2_·2H_2_O	0.040
**Solutions**	**Volume [mL/L]**
Resazurin 1% (*w*/*v*)	1
Vitamin solution	10
Wolfe‘s mineral solution	10
adjust to pH 6.3 with NaOH	

**Table 2:** Composition of the vitamin solution.

Species	Concentration [mg/L]
Biotin	2
Folic acid	2
Pyridoxine-HCl	10
Thiamine-HCl	5
Riboflavin	5
Nicotinic acid	5
Ca-D-pantothenate	5
Vitamin B12	0.1
p-Aminobenzoic acid	5
(±)-α-Lipoic acid	5

**Table 3:** Composition of the Wolfe‘s mineral solution.

Species	Concentration [g/L]
EDTA	0.5
MgSO_4_·7 H_2_O	3
MnSO_4_·H_2_O	0.5
NaCl	1
FeSO_4_·7 H_2_O	0.1
CoCl_2_·6 H_2_O	0.1
CaCl_2_	0.1
ZnSO_4_·7 H_2_O	0.1
CuSO_4_·5 H_2_O	0.01
AlK(SO_4_)_2_·12 H_2_O	0.01
H_3_BO_3_	0.01
Na_2_MoO_4_·2H_2_O	0.01
EDTA	0.5
MgSO_4_·7 H_2_O	3
MnSO_4_·H_2_O	0.5
NaCl	1
adjust to pH 6.5 with KOH	

**Table 4:** Composition of the autotrophic media for *C. carboxidivorans* cultivation.

**Species**	**Concentration [g/L]**
NH_4_Cl	1
KH_2_PO_4_	0.33
K_2_HPO_4_	0.45
MgSO_4_·7 H_2_O	0.1
FeSO_4_·7 H_2_O	0.026
**Solution**	**Volume [mL/L]**
Wolfe’s mineral solution	20
Ni-Se-W stock	1
Resazurin 1% (*w*/*v*)	1
After autoclavation add:	
Wolfe’s vitamin solution	10
L-Cysteine (100 g/L)	5
Na_2_CO_3_ (50 g/L)	20
adjust pH to 6.2 with NaOH	

**Table 5:** Composition of Ni-Se-W stock for the autotrophic media.

Species	Concentration [g/L]
Nitrilotriacetic acid	1.5
NiCl_2_·6 H_2_O	0.6
Na_2_SeO_3_	0.2
Na_2_WO_4_·2 H_2_O	0.2

**Table 6:** Overview of metabolite formation in the individual bioreactor experiments.

		CHER I	CHER II	CHER III	CHER IV
Acetate	max concentration [g/L]	25.8	40.4	46.6	40.3
	max. productivity [mg/L/d]	936	906	910	861
Butyrate	max concentration [g/L]	5.5	11.3	10.67	12.9
	max. productivity [mg/L/d]	73	384	543	710
Caproate	max concentration [g/L]	2.4	5.1	1.7	1.1
	max. productivity [mg/L/d]	196	75	229	91
Ethanol	max concentration [g/L]	0.03	1.0	3.4	1.1
	max. productivity [mg/L/d]	18	132	424	117

## Data Availability

Raw data supporting the conclusions of this article may be made available by the authors on request. Raw sequence data may be found in NCBI’s Bioproject PRJNA1256324.

## Supplementary Materials

The following supporting information can be downloaded at: https://www.mdpi.com/article/10.3390/bioengineering12050470/s1.

## Abbreviations

The following abbreviations are used in this manuscript:

BES	2-Bromoethanesulfonate
BSTFA	O-Bis (trimethylsilyl) trifluoroacetamide
CHER	Chain Elongation Reactor
GCMS	Gas Chromatography Mass Spectrometry
HPLC	High-Performance Liquid Chromatography
OD	Optical Density
SIM	Selected Ion Monitoring
