# Supplementary material for: Enhancing Gas Fermentation Efficiency via Bioaugmentation with Megasphaera sueciensis and Clostridium carboxidivorans
Source: Bioengineering (Basel). 2025 Apr 29;12(5):470. doi: 10.3390/bioengineering12050470 (PMC12109107; doi:10.3390/bioengineering12050470)
Supplement: Supplementary file 1 [file bioengineering-12-00470-s001.zip › bioengineering-3562012-supplementary.pdf]

Supplementary Information

MDPI bioengineering, ISSN (electronic): 2306-5354

Enhancing Gas Fermentation Efficiency via Bioaugmentation with *Megasphaera sueciensis* and *Clostridium carboxidivorans*

Clemens Hiebl [1], Dominik Pinner [1], Hannes Konegger [1], Franziska Steger [1], Dina Mohamed [2] and Werner Fuchs [1]*

[1] University of Natural Resources and Life Sciences, Vienna, Department of Agricultural Sciences, Institute of Environmental Biotechnology, Konrad-Lorenz-Strasse 20, 3430 Tulln an der Donau, Austria
[2] Institute of Chemical Technologies and Analytics, TU Wien, Getreidemarkt 9, 1060 Vienna, Austria
* Correspondence: werner.fuchs@boku.ac

Material & Methods

Metataxanomic analysis

Illumina paired-end MiSeq sequencing was performed by Microsynth AG (Balgach, Switzerland) yielding read lengths of 2x250 bp. Raw sequence data may be accessed via NCBI Bioproject PRJNA1256324.

Metataxonomic profiling was accomplished by using bundled modules of the QIIME2 software suite[1] (qiime2-amplicon-2024.10) with the installed q2-fondue plugin to access reference data (Bioproject PRJNA791644, Steger et al.) from the SRA. For trimming, filtering, denoising and amplicon sequence variant (ASV) inference, the DADA2[2] plugin of QIIME2 was used. Technical and primer sequences were trimmed prior executing the default DADA2 workflow. Taxonomic classification of the retrieved feature information was accomplished involving the classify-sklearn routine and the Greengenes2[3] database (2022-10) of the V4 region. Subsequent filtering of the abundance table and plotting of the microbial community was realized with QIIME2.

Complementary results of metataxonomic analysis

A direct and valid comparison of sequencing samples CHER-1 to CHER-4 in the current study and TB3 (B) from a preliminary study by Steger et al. is not possible, due to the usage of different primer pairs used for amplification of the amplicons. However, as illustrated in supplementary figure S1, the first introduction and manifestation of a member of the *Megasphaeraceae* family, namely *Megasphaera sueciensis*, could be verified exclusively for the samples CHER-1 to CHER-4. Neither *Megasphaera sueciensis* nor *Clostridium carboxidivorans* could be detected in the initial sample TB3 (B).

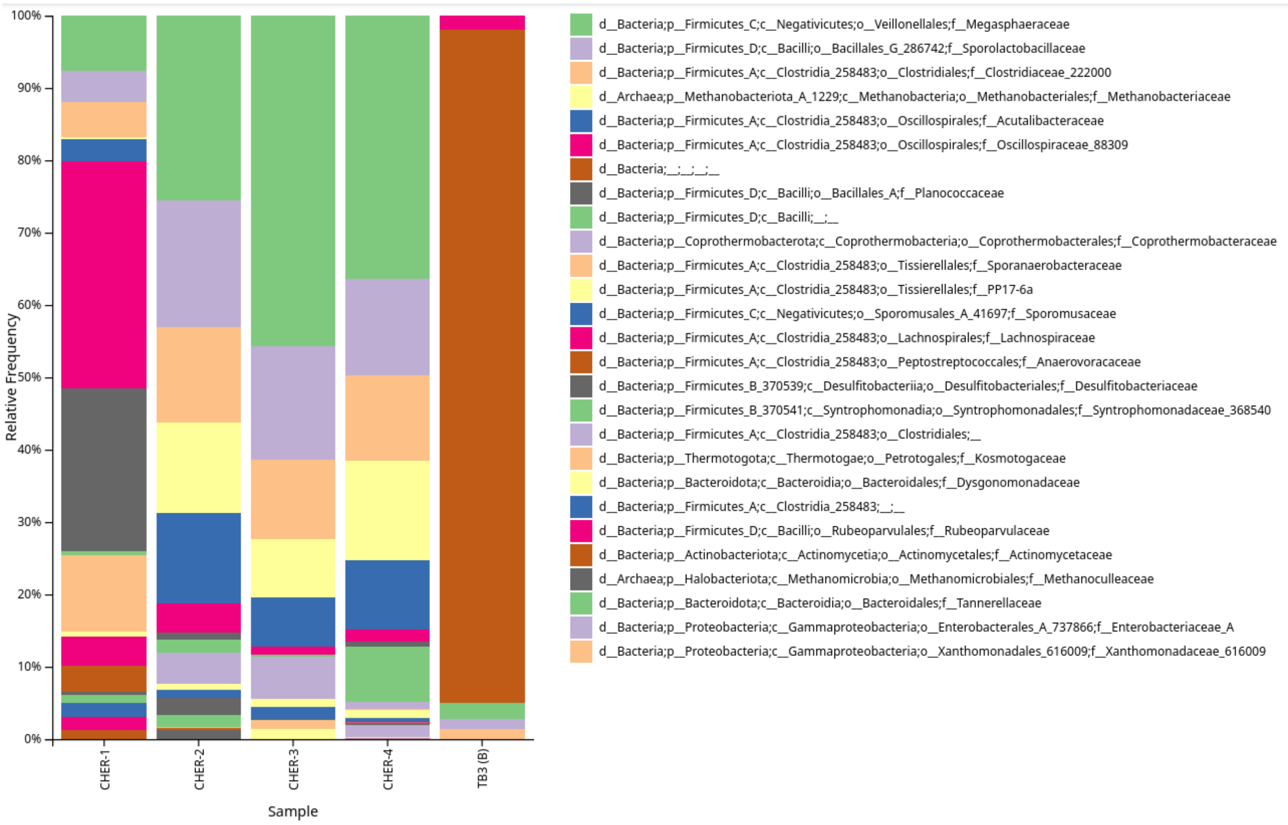

<u>Supplementary Figure S1:</u> Microbial community composition resolved at family level of samples CHER-1 to CHER-4 and the reference sample TB3 (B) of Steger et al.. Microbial taxa with a relative abundance < 1% were excluded from the plot to improve readability.

<u>Primers used for amplification of the V4 region of the 16S rRNA gene</u>

Widely used primers 515F by Parada et al. [4]and 806R by Apprill et al. [5]were used for the amplification of the V4 amplicon according to the cycling conditions outlined in the method section. The sequence of the respective primers is given below, the locus-specific region is shown in bold letters.

V4_Ill_Nextera_fw (515F):
TCGTCGGCAGCGTCAGATGTGTATAAGAGACAGNNNNN**GTGYCAGCMGCCGCGGTAA**

V4_Ill_Nextera_rv (806R):
GTCTCGTGGGCTCGGAGATGTGTATAAGAGACAGNNNNN**GGACTACNVGGGTWTCTAAT**

Sequence Quality Histograms

Sequencing statistics computed by fastqc and reported by MultiQC v1.0 dev [6]for the V4-16S rRNA amplicons of four samples (CHER-1 to CHER-4) shown in supplementary figures S2 and S3.

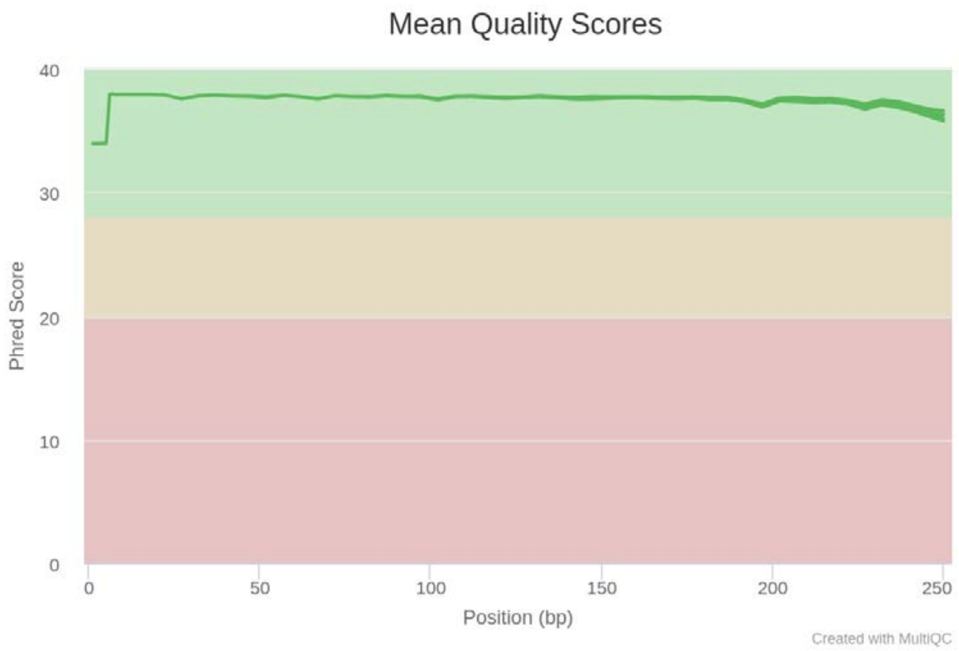

Supplementary Figure S3: Sequence quality histograms of forward reads of samples CHER-1 to CHER-4. A high sequencing quality (Phred score >28) over the entire amplicon could be accomplished for all samples.

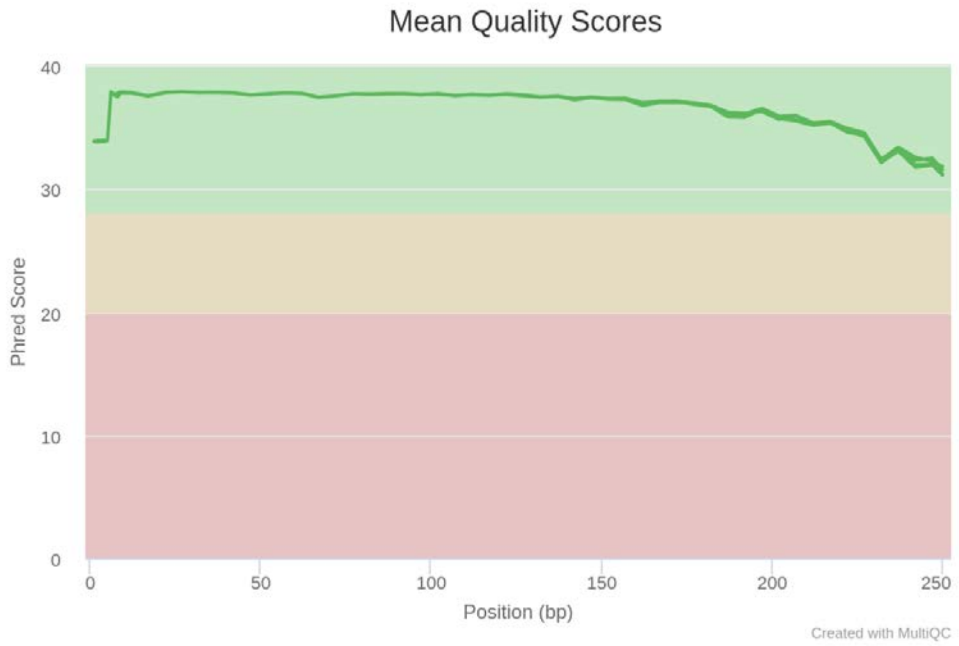

Supplementary Figure S2: Sequence quality histograms of reverse reads of samples CHER-1 to CHER-4. A high sequencing quality (Phred score >28) over the entire amplicon could be accomplished for all samples.

DADA2 amplicon inference statistics (to supplementary figure 1)

The DADA2 software package, was used, as described in the methods section, to infer amplicon sequence variants (ASVs) for the four samples CHER-1 to CHER-4. The following statistics (supplementary figure S4) were computed using the dada2 routine of the QIIME2 software suite (amplicon distribution 2024.10). About 80 % of all raw sequences could be used for the inference of ASVs.

sample-id #q2:types	input numeric	filtered numeric	percentage of input passed filter numeric	denoised numeric	merged numeric	percentage of input merged numeric	non-chimeric numeric	percentage of input non-chimeric numeric
384adbbe-2653-4bb1-9b62-14c4a316a28c	120810	120147	99.45	119146	114282	94.6	99266	82.17
4d53a8f6-e201-455d-b757-36d0aa3a1e5e	113958	113382	99.49	112394	107730	94.53	90508	79.42
a4c76a36-c291-4b7b-b167-66cc410477ac	159195	158261	99.41	157219	151824	95.37	132873	83.47
ec75c71f-42ca-482c-8031-6e836a6d2ff2	168879	168281	99.65	167188	161396	95.57	138503	82.01

Supplementary Figure S4: *Inference statistics of samples CHER-1 to CHER-4 (all anonymized with hash ids to prevent analysis bias). After filtering, denoising and removal of chimeric sequences, a minimum of >90.000 sequences for subsequent analyses could be retrieved for all considered.*

ASV rarefaction curves

To assess, if sufficient DNA sequencing depths have been reached in the sequencing campaign for the samples CHER-1 to CHER-4 and reference sample TB3 (B) (https://trace.ncbi.nlm.nih.gov/Traces?run=SRR17317690), an alpha-rarefaction analysis was conducted with the QIIME2 module qiime diversity alpha-rarefaction using the parameters: *--p-steps 16*, *--p-min-depth 10* and *--p-max-depth 50000*. Both supplementary figures S5 and S6 indicate, that the obtained number of sequences was sufficient to faithfully describe the microbial communities

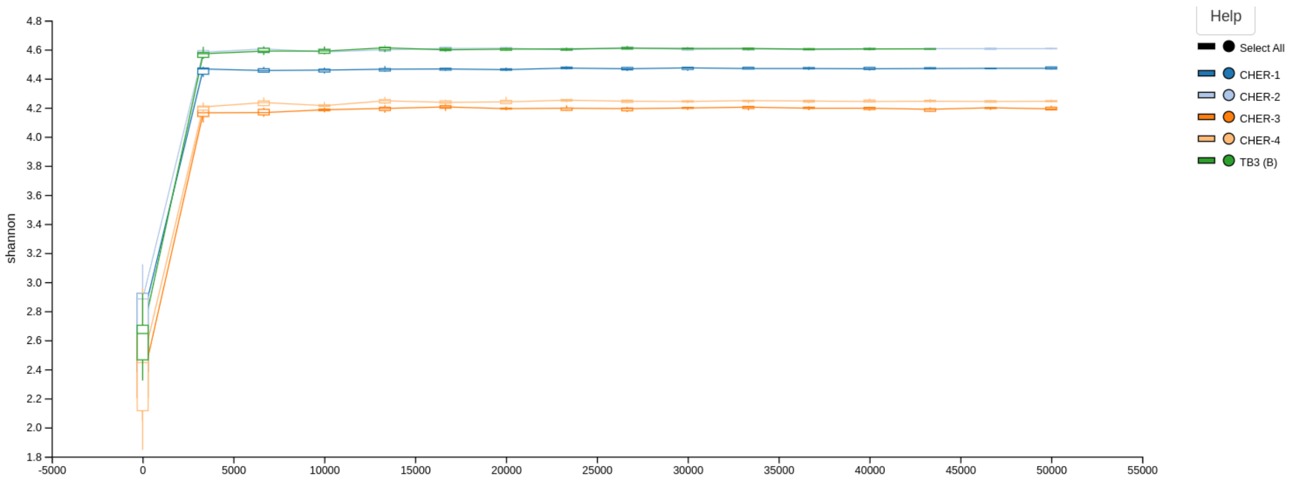

Supplementary Figure S5: *Boxplot diagram showing the computed Shannon index against various levels of sequencing depth. Convergence of this metric could be observed after the sequencing of approximately 8.000 sequences for each sample.*

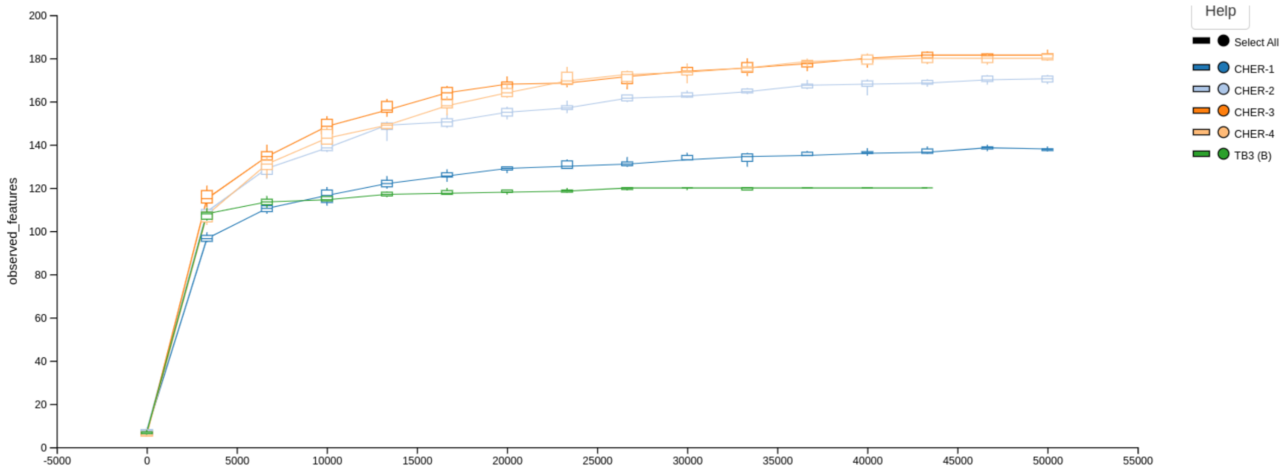

<u>Supplementary Figure S6</u>: Boxplot diagram of observed features (=ASVs) at various levels of sequencing depth. Convergence could be observed after the sequencing of > 40.000 sequences for

<u>Confidence of taxonomic classifications:</u>

The taxonomic classification of inferred features (=ASVs) was conducted with the QIIME2 module qiime feature-classifier classify-sklearn and the Greengenes2 *2022.10.backbone.v4.nb.sklearn-1.4.2.qza* classifier, accessed and obtained via https://library.qiime2.org/data-resources

Neither *Clostridium carboxidivorans* nor *Megasphaera sueciensis* ASVs were detected in reference sample TB 3 (B). For the species *Clostridium carboxidivorans*, no corresponding inferred ASVs could be detected in CHER-1 to CHER-4, implying that the augmented bacterium did not manifest in the given microbial community. A systematic failed amplification of the 16S rRNA V4 region of *C. carboxidivorans* with the given primers 515F and 806R due to insufficient annealing may be excluded, as supported by a study of Feliu-Paradeda et al. [7].

For *Megasphaera sueciensis* however, four matched corresponding features (three resolved at species level), contained in samples CHER-1 to CHER-4, could be assigned with prediction confidences of > 0.97, as illustrated in the supplementary figure 7.

Feature ID #q2:types	Taxon categorical	Confidence categorical
28729021c9a1eeae1270896d184e2552	d__Bacteria; p__Firmicutes_C; c__Negativicutes; o__Veillonellales; f__Megasphaeraceae; g__Megasphaera_A_38685; s__Megasphaera_A_38685 sueciensis	0.9820378249056454
6aec442e6d64815666284a494d17af1e	d__Bacteria; p__Firmicutes_C; c__Negativicutes; o__Veillonellales; f__Megasphaeraceae	0.9999997736467059
7ddc6ffb9c0e6ee1010eae7a249c4a08	d__Bacteria; p__Firmicutes_C; c__Negativicutes; o__Veillonellales; f__Megasphaeraceae; g__Megasphaera_A_38685; s__Megasphaera_A_38685 sueciensis	0.9760708366818451
904b43d418414a5b42dfbfce9959519b	d__Bacteria; p__Firmicutes_C; c__Negativicutes; o__Veillonellales; f__Megasphaeraceae; g__Megasphaera_A_38685; s__Megasphaera_A_38685 sueciensis	0.9767123728865745

<u>Supplementary Figure S7</u>: Inferred features (=ASVs) with corresponding matches for the family Megasphaeraceae in the Greengenes2 16S rRNA database. Megasphera sueciensis was richly abundant in samples CHER-1 to CHER-4, however not present in the reference sample TB3 (B).

The presented data suggests, that *Megasphaera sueciensis* successfully integrated into the microbial communities of the characterized samples CHER-1 to CHER-4.

References

1. Bolyen, E. *et al.* Reproducible, interactive, scalable and extensible microbiome data science using QIIME 2. *Nat. Biotechnol.* **37**, 852–857 (2019).
2. Callahan, B. J. *et al.* DADA2: High-resolution sample inference from Illumina amplicon data. *Nat. Methods* **13**, 581–583 (2016).
3. McDonald, D. *et al.* Greengenes2 unifies microbial data in a single reference tree. *Nat. Biotechnol.* **42**, 715–718 (2024).
4. Parada, A. E., Needham, D. M. & Fuhrman, J. A. Every base matters: Assessing small subunit rRNA primers for marine microbiomes with mock communities, time series and global field samples. *Environ. Microbiol.* **18**, 1403–1414 (2016).
5. Apprill, A., Mcnally, S., Parsons, R. & Weber, L. Minor revision to V4 region SSU rRNA 806R gene primer greatly increases detection of SAR11 bacterioplankton. *Aquat. Microb. Ecol.* **75**, 129–137 (2015).
6. Ewels, P., Magnusson, M., Lundin, S. & Käller, M. MultiQC: Summarize analysis results for multiple tools and samples in a single report. *Bioinformatics* **32**, 3047–3048 (2016).
7. Feliu-Paradeda, L., Puig, S. & Bañeras, L. Design and validation of a multiplex PCR method for the simultaneous quantification of Clostridium acetobutylicum, Clostridium carboxidivorans and Clostridium cellulovorans. *Sci. Rep.* **13**, 1–12 (2023).
